# Qualitative Insights on Preventive Group Training in LTC Facilities: Key Influencing Factors

**DOI:** 10.1111/1460-6984.70226

**Published:** 2026-03-19

**Authors:** Isabell Fesser, Klaus Hager, Simone Miller, Wenke Walther

**Affiliations:** ^1^ Institute of General Practice and Palliative Care Hannover Medical School Hanover Germany; ^2^ Experimental Phoniatrics at the ENT‐clinic Hannover Medical School Hanover Germany

**Keywords:** evaluation, group processes, nursing home, old age, prevention, qualitative research

## Abstract

**Background:**

The cluster randomised controlled trial OrkA (Orofaciopharyngeal and linguistic‐communicative activation in older age) aims to evaluate the efficacy of a preventive group programme for older adults in long‐term care (LTC) facilities. The programme objective is to support the maintenance of swallowing, speech and communication skills in later life. Group therapy and preventive services are two options that could help to counteract the current shortage of specialists and long waiting times for speech and language therapy (SLT). However, these strategies are rarely chosen to meet outpatient SLT needs in Germany. Group interventions in LTC facilities for older adults are diverse and have already been researched in various areas. Group interventions that promote language and communication in LTC facilities lead to an increase in well‐being. However, group interventions delivered by speech and language therapists in LTC facilities are topics that have received little research attention to date.

**Aims:**

The present evaluation was conducted to investigate: (1) the conditions required for a successful group intervention in an LTC setting, and (2) the skillset needed by trainers.

**Methods and Procedures:**

The study involved multiple stakeholders: LTC facilities (including staff and residents) and speech and language therapists (as group trainers). Seven semi‐structured interviews were conducted with four group trainers following each intervention. Both the transcription and the qualitative content analysis based on Mayring's approach were carried out using MAXQDA. Categories were developed both deductively and inductively.

**Outcomes and Results:**

The success of preventive intervention programmes for older adults in LTC settings is influenced by external and internal factors. External factors include the institutional conditions of the facilities, room environments and the effectiveness of communication among the different stakeholders (nursing staff, trainers, project administration, residents). Internal factors comprise group characteristics and dynamics, participants limitations and group‐related challenges. This interplay necessitates a comprehensive trainers’ skill‐set, including: (1) disruption management, (2) manual‐based programme implementation and adaptation, (3) empathic and appreciative behaviour, (4) self‐reflective competencies, and (5) effective facilitation of group processes.

**Conclusion and Implications:**

Preventive measures concerning swallowing, speech and communication abilities among older adults in LTC facilities must consider a range of critical conditions. External and internal factors are interdependent and should not be considered in isolation. The trainer's role is pivotal, requiring attentiveness to training objectives, external support structures and the individual needs of group members. Leading a group differs markedly from conducting individual therapy, demanding a wide array of trainer competencies, encompassing methodological, interpersonal and personal skills. Thus, group‐based approaches should be incorporated into SLT training programmes.

**WHAT THIS PAPER ADDS:**

*What is already known on this subject*

- Although prevention measures and group offer can counteract the growing shortage of skilled workers, such programmes are rarely used in outpatient speech and language therapy settings in Germany. In 2023, only 0.2% of speech therapy services were provided in group therapy. There are several reasons for the underuse of group offers. These relate to organisational and financial factors. Other reasons may include speech and language therapists not feeling adequately prepared. Therefore, further evaluation of group therapy settings is required. Especially in combination with prevention it is relevant to evaluate the factors that contribute to success. This may be because speech and language therapists do not feel adequately prepared. In psychology and pedagogy, group phases are known that necessitate management and supervision by group leaders.

*What this study adds to existing knowledge*

- For further insight into group formats within SLT, a preventive group training aimed at maintaining swallowing, speech and communication skills for older adults in long‐term care (LTC) facilities was evaluated. The present findings revealed several factors that influence the implementation of preventive group training for the target group. These factors emphasise the importance of trainers possessing specific methodological and social‐communicative competencies to effectively address various internal and external influences.

*What are the potential or actual clinical implications of this work?*

- Group offers are greater than the sum of their parts. As the group itself is an effective factor, its composition is also important. Trainers and therapists need a wide range of skills to handle different situations. While therapists' knowledge and skills concerning group management are highly relevant, they are not necessarily covered in the training curriculum.

## 1 Introduction

In the face of an ageing population, which is creating an increasing demand for care, there has been an increase in the number of individuals being placed in nursing homes. An estimated 5.7 million people in Germany required care in 2023. Of those people, 14% were accommodated in long‐term care (LTC) facilities (Statistisches Bundesamt (Destatis) 2026). Maintaining various abilities to preserve a degree of autonomy is an increasingly important focus of care, particularly in light of age‐related deterioration. However, this is difficult to achieve given the current shortage of skilled workers.

Group therapy setting as well as preventive measures are two of several options for dealing with long waiting times and a shortage of speech therapists (Leder 2025). In Germany, group‐based speech and language therapy (SLT) interventions are included in the guidelines of therapeutic remedies and can be prescribed for all disorders except rhinophonia and dysphagia therapy (HMK digital 2024). However, group therapy and preventive measures are rarely employed in outpatient care in Germany (Leder 2025). In 2023, only 0.2% of speech therapy services were provided in group therapy (WIdO wissenschaftliches Institut der AOK 2024).

Group‐based interventions in SLT are well established in areas of adult interventions. For example, reviews provide initial evidence of the positive effects of group interventions in primary progressive aphasia (PPA) (Watanabe et al. 2024) or in rehabilitative contexts following a stroke or traumatic brain injury (TBI). For both, group approaches have been found to be effective in improving quality of life, communication and language (Greiner et al. 2025).

Beyond its therapeutic benefits, it also provides patients the opportunity to engage with others with similar experiences of speech and communication difficulties, thereby cultivating a sense of community and reducing anxiety in these settings (Weng 2024). The format also offers particular benefits for older adults, as it fosters interaction and communication among individuals within a similar age group (Baron et al. 2018; Hoppmann and Kessler 2024).

Principally, group offers for older adults in LTC facilities are an important area of research. Studies focus on topics such as cognitive stimulation therapy (Galdino De Oliveira et al. 2014), music therapy (Lin et al. 2022), or behavioural interventions, with a particular focus on communication (Davis et al. 2022). It is emphasised that publications on interventions carried out by sSLTs in LTC facilities are rare (Davis et al. 2022). Implementation science research also examines the factors that hinder or facilitate the implementation of group programmes in LTC facilities for older people (Colón‐Emeric et al. 2016; Fisher et al. 2025; Rasing et al. 2025).

As group interventions conducted by SLTs in LTC facilities are a relatively unexplored field (Davis et al. 2022), there is also a lack of evaluations on the realisation of such programmes. To this end, qualitative research must be conducted and, above all, important stakeholders such as participants and trainers should be involved (Skivington et al. 2021)

It is imperative to incorporate trainers/therapists in the evaluation process, given that the German speech therapy training curriculum does not encompass any dedicated content for group therapy (Bundesamt für Justiz 1980). In contrast, group therapy is well‐established in psychotherapy, where guidelines exist to address key aspects such as experience, attitudes towards the group, self‐reflection regarding personal anxieties and collegial exchange (Münch 2024). In SLT, it is insufficiently clear which group interaction or group dynamics skills are required for group‐based programmes. In addition to the financial and organisational reasons (Leder 2025), this may also lead to obstacles when conducting group therapy sessions.

To this end, a preventive intervention programme aimed at maintaining swallowing, speech and communication abilities in older adults living in LTC facilities was evaluated to provide further insight into group formats within SLT for older adults. In addition to a participants' satisfaction survey, which is discussed in another article (Walther and Fesser 2025), the trainers' experiences were considered particularly important in the process evaluation. This intervention formed part of a cluster randomised controlled trial (RCT) known by the acronym OrkA (Orofaciopharyngeal and linguistic‐communicative activation in older age). A total of 14 elderly care facilities in the Hanover region participated in the intervention study. These facilities were categorised as either privately run (*n* = 12), non‐profit (*n* = 10), or owned by the municipality (*n* = 1). They varied in size, with an average of 103 beds (range 36–201), 52 nursing staff (range 6–100) and seven social services staff (range 0–15). A total of 103 residents were included in the study. The majority were female (80.6%) with an average age of 83 (±8.1). The residents had an average care level of 3 (range 0–5) and were only included if they had nor or mild cognitive impairment as determined by the Dementia Detection Test (total score > 8 points) (Kalbe et al. 2004). The group intervention took place over 12 weeks, with the participating residents attending twice a week for 60 min. Each session followed the same pattern: It began with a short mobilisation exercise to prepare participants for subsequent exercises followed by an orofacial sequence involving extra‐ and intraoral movements and exercises to strengthen the muscles of the lips, cheeks, tongue, throat, velum and larynx. Some of these exercises required the use of additional devices such as spatulas, buttons or beads. After the swallowing sequence, a longer section of semantic exercises followed. These included semantic feature analysis and word retrieval exercises, among others. Then there was a communicative sequence that was thematically related to the semantic exercise or determined by the participants in terms of content. Finally, the trainer sang well‐known songs with the group. More details of the study design, recruitment procedures and intervention protocol are provided in the published study protocol (Walther et al. 2024). The trainers were four SLTs (aged 28–39 years), who worked on the OrkA project. Three of them held a master's degree and one a bachelor's degree. The team comprised one trainer with experience in neurorehabilitation and group therapy, one with experience in children's group therapy and one with expertise in voice training groups. During the study, three of the trainers led multiple groups, allowing them to develop a range of skills across different settings. The present qualitative evaluation focused on trainer‐ and group‐related factors, as well as external influences that either supported or impeded the intervention. Specifically, it sought to address the following research questions.

### 1.1 Research Questions

1. What conditions are necessary for a successful group intervention with older adults in an LTC facility?
2. Which factors promote or hinder the success of such a group intervention?
3. What skills are required of trainers to successfully deliver such a group intervention?

## 2 Materials and Methods

A qualitative interview study was conducted with the four trainers who had delivered the group intervention to older adults in LTC facilities, to gather detailed insights into the trainers’ experiences. Semi‐structured qualitative interviews offer a comparison approach, balancing a clear framework with the flexibility for open‐ended narration and in‐depth questioning (Döring and Bortz 2016). However, interviewers must possess the necessary experience and professionalism to respond sensitively to participants’ responses, thereby encouraging open expression. Moreover, they should employ effective follow‐up questions to elicit responses that are both deep and broad across the relevant topics (Krell and Lamnek 2010).

Development of the semi‐structured interview guide was based on WW's professional experience in previous studies (Müller‐Mundt and Walther 2022) and the overarching research interest in evaluating the intervention programme. To address the relevant themes in relation to the research questions (Siering et al. 2002), the interview guide collected information on general experiences (e.g., initiation of the intervention, institutional support, trainers’ concerns and reflections) and the implementation process (e.g., issues with materials, exercises or group dynamics). The semi‐structured interview guide (see Supporting Information S1) was pilot tested by WW and SM prior to its use with the four trainers. The first two interviews were conducted together for training purposes.

### 2.1 Data Collection

As the trainers were already part of the study team, there was no need to recruit them separately. Invitations were sent via email and in person. All participants were informed about the voluntary nature of their participation, the study objectives and the data protection protocol, and written informed consent was obtained. The study was conducted in accordance with the Declaration of Helsinki. All interviews were carried out shortly after the end of each intervention. Seven interviews were conducted in total, covering 11 intervention instances, with an average duration of 55 min (range: 39–112 min). These interviews took place between December 2023 and March 2025. They occurred in quiet meeting rooms at the research institute and were recorded as audio files. A maximum of two experienced female researchers with doctoral qualifications (WW, SM) carried out the interviews using the semi‐structured interview guide. One trainer managed multiple groups simultaneously and therefore reported on two groups during each interview. Additionally, a tandem interview was conducted with two trainers simultaneously. Apart from a notetaker during the tandem interview, no one else was present during the interviews. Field notes were also made by the interviewers. Further information about the interviews, including the number of trainers and researchers present, can be found as Supporting Information S2.

### 2.2 Data Collection Limitations

The trainers who carried out the intervention were also members of the scientific research team. As only the trainers were interviewed, rather than other stakeholders such as employees from the care homes, the survey was limited in scope. These two aspects may have led to selection bias. Additionally, one of the trainers may have evaluated the interview material in a manner that confirmed her own views, potentially leading to confirmation bias. Interviewer bias may have been introduced by the fact that, in some interviews, both interviewers conducted the interview, while in others, only one interviewer was present.

### 2.3 Data Analysis

Transcription marked the first phase of the analysis process. Interviews were transcribed verbatim and subsequently adapted to written language conventions. The transcripts followed the method of Kuckartz and Rädiker (2022) and were created using MAXQDA software (version 24.7.0). Non‐verbal cues such as ‘laughing’ and extended pauses were noted in parentheses, and timestamps were added at the end of each paragraph. Emphasised and incomprehensible words were marked accordingly. Interviewees were anonymised as Trainer 1 (T1) through Trainer 4 (T4) across the transcripts. Given the tandem interview with T1 and T2, and repeated interviews with three of the trainers (T1, T3, T4), interview instances were denoted accordingly (e.g., I1, T2 = interview 1 with trainer 2; I5, T3 = interview 5 with trainer 3). Transcripts were not returned to participants for comment or correction.

Data analysis was conducted by IF using a parallel form of the qualitative content analysis based on Mayring (2022). This approach involved the development of deductive and inductive categories. Deductive main and subcategories, which were derived from the interview guide, are based on the Medical Research Council's guidelines (Skivington et al. 2021). These refer to contextual factors (e.g., facilities), implementation (e.g., manual adherence) and interest group involvement (e.g., trainers and participants) (Skivington et al. 2021). The inductive categories were formed through subsumption. Therefore, the material was reviewed in accordance with predefined selection criteria (Mayring 2022). Inductive category formation referred to important aspects of the interview material that were not included in the deductive categories. New categories were either formed or the material was assigned to an existing category. Definitions were established for all categories, and coding and distinction rules were developed where necessary. The categories were revised after 10%–50% of the material has been processed (Mayring 2022). In this regard, IF has expanded the category system to include inductive categories, that is, new but relevant topics. Category definitions, anchor examples and coding rules were provided for categories that were difficult to distinguish from one another. Further information on category development can be found in the coding tree, which is available as Supporting Information S3. This document contains the definitions, theories and research questions relating to the categories and their development. Transcripts were segmented into coding units and assigned to the most suitable categories. To reduce potential bias, sample coding on approximately 20% of the total interview material was carried out independently by two researchers (WW and IF). This was achieved through the extensive tandem interview (I1), during which two group trainers (T1 and T2) were interviewed simultaneously. The first trial coding was based on the initial version of the coding tree, which contained deductive and inductive categories with definitions for the main and subcategories. Any observed consistencies and discrepancies were recorded quantitatively in a comparison. WW and IF then discussed any inconsistencies, and refined definitions or changed subcategories as necessary. This revision supported the final completion of the coding process for the qualitative analysis. Any discrepancies were discussed and codes were subsequently re‐evaluated. Inter‐coder reliability was initially 67.9% and improved to 97.1% following revision. The coding sample that was carried out after the revision was performed by the same two researchers (WW and IF). IF then finalised the category definitions. Once all code units were categorised in MAXQDA, representative anchor examples were selected for each category.

## 3 Results

**FIGURE 1:**
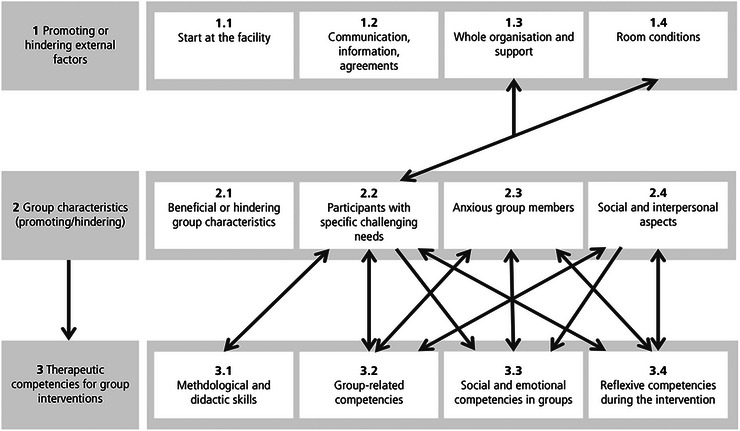
Presentation of the main and subcategories, and the influence of the categories on each other.

This article presents the findings across the following three main categories: (1) promoting or hindering external factors, (2) group characteristics (promoting/hindering) and (3) therapeutic competencies for group interventions. Each main category is further divided into four subcategories. The first main category was derived deductively from the interview guide. The second and third main categories were derived inductively from the material. Further information on the deductive and inductive development of the main and subcategories can be found in the coding tree (see Supporting Information S3). The third category ‘Group characteristics’ acted as a nexus between external and internal factors, since trainer‐related competencies were largely developed in response to the specific needs of each group (see Figure 1).

These three overarching categories encompass the key factors influencing the success of group interventions with older adults in LTC facilities. The first category pertains to external conditions experienced by trainers upon arrival and throughout the intervention. The second focuses on group dynamics and participant‐related aspects. The third category addresses internal, trainer‐centred factors, including therapeutic competency and group challenges.

### 3.1 Promoting or Hindering External Factors

External factors identified as influential included the initial reception at the LTC facility, communication and agreements among stakeholders, the overall organisation and level of support, and the physical conditions of the rooms in which the interventions took place.

#### 3.1.1 Start at the LTC Facility

Trainers initially commented on the reception they received at the LTC facility. Some reported a welcoming, organised and supportive environment upon arrival, which facilitated a smooth beginning to the intervention. They noted that staff were informed of the programme start date and offered guidance and orientation upon entry:

And as I said, it was really nice to be there for the first time, because there's a reception desk right there, where people knew directly where I was supposed to go, and yes, I felt well looked after. (I5, T3)

Conversely, other trainers described less favourable experiences, including confusion and a lack of preparation on the part of the facility, which hindered the initial session:

Um, yes, the people at the front desk seemed rather reserved at first. I asked on my first day which room I could go to, and introduced myself and explained again what I wanted to do […] but it wasn't clear where I was from, what I was doing. It wasn't clear which room we were allowed to go to, either. (I3, T4)

#### 3.1.2 Communication, Information and Agreements

The trainers also discussed various aspects of communication and agreement among the parties involved (i.e., LTC facility staff, trainers, participants). Trainers who encountered an unfavourable reception frequently observed that participants had not been informed about the intervention's commencement. Some participants were entirely unprepared for the session and consequently felt ill‐equipped for the nature of the appointment. This lack of preparation may have caused a degree of discomfort and potentially contributed to a higher rate of drop‐out:

Unfortunately, the participants weren't informed either, they were totally shocked. They had no idea what was going on. (I3, T4)

Trainer T3 reported a similar experience with her second group. To avoid overwhelming the participants, she independently decided to reschedule the first appointment. The following positive account of the rescheduled session highlights the beneficial outcome of her decision:

The only thing was that the residents didn't know about the first appointment […] but then after I had explained again, also with the superior, the second appointment, which was actually our first one, went really well. (I5, T3)

In addition, scheduling overlaps between the intervention and other activities offered by the institution sometimes forced participants to choose between them:

Especially on Friday, when there were some really attractive offers at the facility, that they really wanted to go to. I mean, they were free to do so, but they were all very disciplined and still came to me a lot anyway. (I6, T4)

#### 3.1.3 Whole Organisation and Support

The overall organisational structure and level of support available significantly influenced the implementation of the preventive intervention. An optimal structure was one in which the intervention was seamlessly integrated into the weekly activity schedule, becoming an inherent part of the institutional framework. A key factor identified was the breadth of support offered by the facility, encompassing the provision of materials, personnel and institutional backing, as well as the management of disruptions to enable uninterrupted sessions. In some facilities, nursing staff provided extensive assistance to participants—for instance, helping with voluntary tasks or homework between sessions and ensuring timely attendance in the designated room. Additionally, some LTC facilities demonstrated considerable cooperation in the supply of requested items such as water, small spoons, juice and straws:

And yes, water was also provided during the tests, so whenever I needed something, ‘Could you arrange it somehow like this’ or ‘I need some more glasses’, it was no problem at all. (I5, T3)

In contrast, other facilities struggled to ensure that participants with substantial care needs arrived punctually at the group room. As a result, not all participants could begin the weekly session on time, leading to interruptions that trainers had to manage:

It was also partly because they just arrived later and then someone else joins in and wants to know what's happening, seems a little impatient, so then the task is interrupted again. (I6, T4)

Moreover, last‐minute changes to the scheduled times (e.g., delays in the start time) required trainers to act swiftly to maintain adherence to the weekly programme:

And the time could stay the same, because I think it was going to be changed, but then most of the residents wouldn't have been able to make it, which means I might have lost some of them for organisational reasons. (I1, T1)

#### 3.1.4 Room Conditions

Optimal room conditions were described as bright, spacious, clean, well‐organised and suitably equipped for training or meeting purposes, with rooms reserved specifically for these functions. By contrast, suboptimal rooms were characterised as dark, cramped, cold, untidy or inadequately prepared and reserved for training needs. While some trainers were consistently allocated a comfortable and orderly room, others had to change locations frequently. In such cases, trainers were often responsible for cleaning and preparing the room prior to participant arrival, including adjusting the temperature to ensure a comfortable environment:

Indeed, it was more a case of arriving a little early because you had to warm up the room somehow, which wasn't always possible in such a small space […] because that's important for the well‐being of the participants, who simply can't sit in a cold room for an hour. Exactly, yes. (I1, T1)

Exactly, in the other group it was like that, I actually went there a little earlier, because the room was very small, so sometimes I cleared a few things away because it was used as a bit of a storage room. (I1, T1)

**FIGURE 2:**
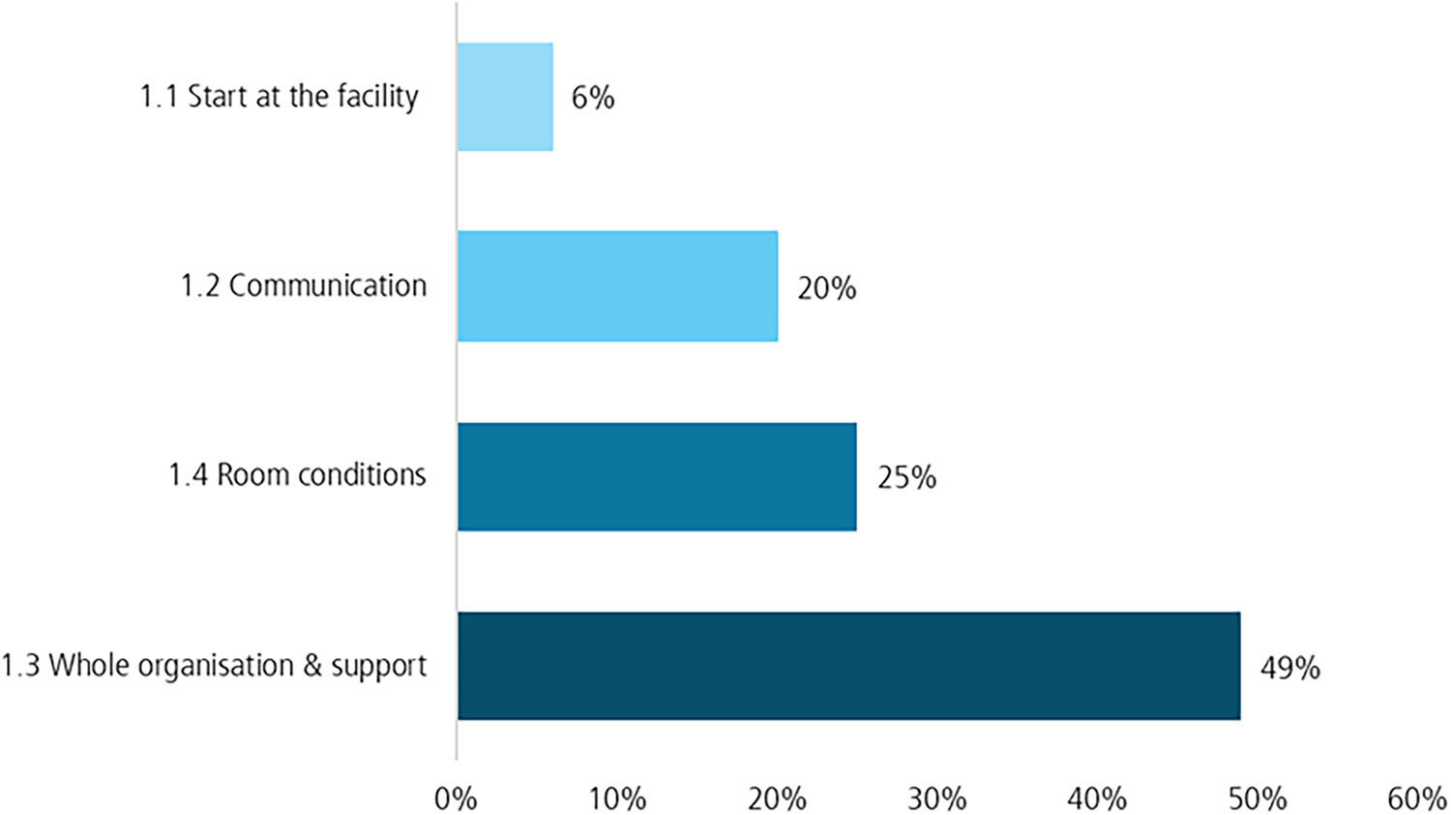
Code‐frequency of coding units for the subcategories of the first main category.

As shown in Figure 2, the categories were coded at different frequencies. It demonstrates that the room conditions, communication aspects, and the organisational support were particularly important to the trainers.

### 3.2 Group Characteristics (Promoting/Hindering)

Group characteristics served as a nexus between external and internal factors, as trainer‐related competencies were largely developed in response to group‐specific needs. Trainer accounts highlighted both supportive and challenging group dynamics. Several trainers also identified participants with particular needs (e.g., individuals displaying heightened anxiety), alongside a variety of interpersonal and social dynamics.

#### 3.2.1 Beneficial or Hindering Group Characteristics

One commonly reported hindrance was a lack of homogeneity within groups, particularly regarding participants’ physical and cognitive capacities:

Now I'll stick with the second group, because there were actually more difficulties there, um, because with one or two of them, either cognitive impairments, slight cognitive impairments were already noticeable, um, or a certain form of frailty was very prominent, that is, in the group I had to address such limitations more. (I1, T1)

However, some groups were characterised by beneficial traits, such as intrinsic motivation and a strong interest in the study content and preventive goals. Nevertheless, the programme demands required trainers to exhibit domain‐specific expertise:

Yes, some of the participants asked some very profound questions, so they had really thought about the content, which they were able to grasp well, and asked some more complex questions. They weren't shy at all. Every week, there were new questions about general topics or personal problems related to swallowing or finding the right words. (I1, T2)

#### 3.2.2 Participants with Specific Challenging Needs

Certain group members presented specific challenges, including physical or cognitive impairments, degenerative illnesses (e.g., Parkinson's disease), inattentiveness or a highly critical attitude:

But she sometimes complained about it, even in a humorous way, but that just makes it difficult. Like, if it's actually her fault, because she maybe wasn't paying attention or didn't notice. (I5, T3)

#### 3.2.3 Anxious Group Members

Participants experiencing significant anxiety exhibited varying reasons for their apprehension. In some cases, anxiety stemmed from the intervention content, while in others it related to pre‐existing difficulties, such as disorientation in time and space or social discomfort in group settings:

It actually started right at the beginning, when she said: ‘Yes, I never feel comfortable, I'm always afraid of going into group situations because I can't find my place there. (I7, T1)

#### 3.2.4 Social and Interpersonal Aspects

Groups with poor social dynamics (e.g., reduced mutual understanding, heightened potential for conflict, destructive criticism) required trainers to place a greater emphasis on fostering interpersonal competence:

And the others were more like outliers, so afterwards some people said, ‘I wondered how they were even accepted into the program’, and then I thought, well, there are guidelines and inclusion criteria for that. (I5, T3)

In contrast, positive interpersonal relationships within the group could significantly enhance the intervention experience:

I think that's always a really important question and I can only answer it positively because the three other women, especially one of them, are very appreciative. (I7, T1)

**FIGURE 3:**
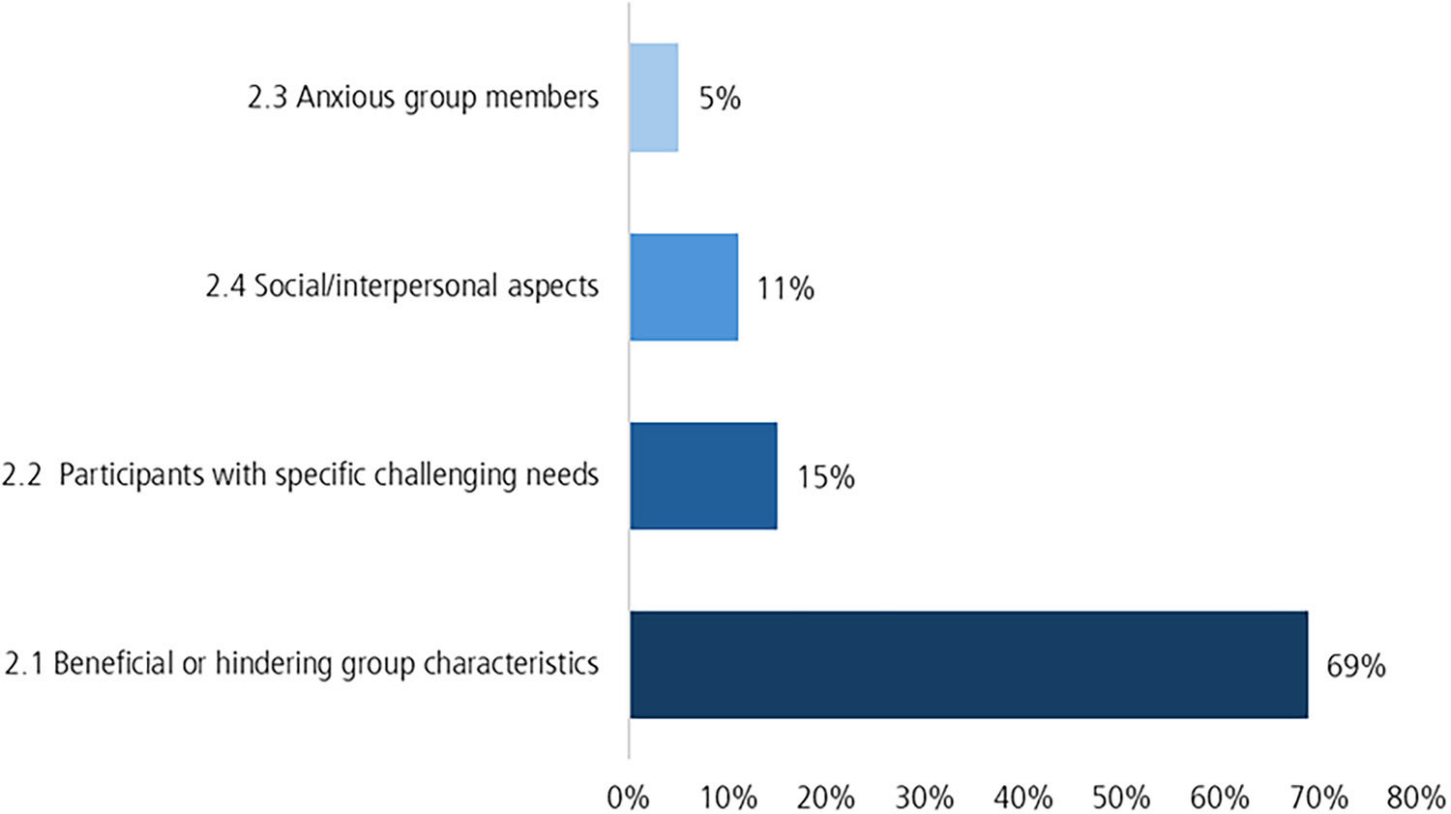
Code‐frequency of coding units for the subcategories of the second main category.

As illustrated in Figure 3, the majority of codes were assigned to group characteristics. Indeed, the interview data contained a great amount of information about group members with specific challenging needs.

### 3.3 Therapeutic Competencies for Group Interventions

This section addresses trainers’ personal and professional development throughout the intervention. During this period, trainers were required to cultivate a range of competencies, including methodological problem‐solving abilities, didactic skills, group‐related competencies, social and emotional awareness within group contexts, and reflexive capacities. A high level of empathy and sensitivity proved essential throughout the process.

#### 3.3.1 Methodological and Didactic Skills

Methodological and didactic competencies pertained primarily to exercises conducted within the preventive intervention sessions. These abilities were particularly important when exercises were either too challenging or insufficiently stimulating for the target group, or when the materials did not meet participant expectations. Methodological problem‐solving skills focused on adaptation of the swallowing and speech‐related exercises. In situations where tasks were deemed unsuitable, unfeasible or rejected by participants, trainers required a robust understanding of the objectives, target musculature and underlying mechanisms of each exercise to propose appropriate alternatives:

Yes, so that they simply practiced the same anatomical structure but with an alternative task, if possible. Sometimes it was easy to modify, right? (I4, T1)

Didactic skills were more closely related to the instructional components of the sessions. Trainers needed to effectively explain the relevance of each exercise, often incorporating demonstrations and repetition to ensure participants could understand and apply them independently—particularly for homework tasks:

I then explained something again, even though I may have already explained it before. I just explained more about the muscles and how they are structured in the face so that they could understand it better, and that was also important for the group. (I3, T4)

#### 3.3.2 Group‐Related Competencies

It was important for trainers to involve all group members in communicative sequences. This competency—encompassing group control—included managing group dynamics and cohesion, adhering to conversational norms, fostering group interactions, demonstrating empathetic responding and constructively addressing tensions and conflicts. Several trainers highlighted the need to monitor the frequency and quality of participants’ verbal contributions during sessions. To ensure equitable participation, they were required to encourage individuals less inclined to speak, thereby maximising the benefits of the intervention for all members. An appreciative approach was therefore vital, respecting the limitations of each participant:

To somehow bring the ladies and gentlemen out of their shell a little bit, who were very shy. Some people wanted to talk a lot, and you had to make sure that things remain balanced and that no one is left out. (I1, T1)

In addition, when working with groups experiencing social or personal conflict, it was necessary to establish a shared framework for discussion, including ground rules for interaction, collective values and mutual respect:

But of course, something like rules of conversation, how do we actually want to operate here as a group? What are our values? What should be the consensus here in the group? How do we want to interact with each other? (I1, T1)

#### 3.3.3 Social and Emotional Competencies in Groups

To respond effectively to interpersonal and group‐dynamic challenges, trainers needed to foster social and emotional competencies within groups. These included sensitivity to group dynamics, heightened awareness and the ability to provide emotional containment. The following excerpt illustrates trainers’ awareness of evolving group dynamics over the course of the intervention:

Initially, there were, you could say, two groups of friends and one individual who participated, but over time they all became closer, and they repeatedly emphasised, that they, um, yes, had got to know each other better and were very happy about that. (I1, T2)

This category encompassed situations requiring acute awareness, appreciation and empathy. For instance, trainers needed to be alert to participants’ discomfort with certain activities, as their ability to perceive such subtleties was essential for effective facilitation and intervention:

But when I've noticed, they feel uncomfortable somehow or find it strange or something, or they look at the other participants as if seeking help, I repeated the explanation. (I3, T4)

Given the vulnerability of the participant group, emotionally charged situations (e.g., death of a fellow resident, expressions of deep self‐doubt) had to be acknowledged and processed sensitively. This capacity may be referred to as emotional containment within groups. It was therefore crucial for trainers to provide adequate time and space for the articulation of emotions, feelings and communicative needs. Moreover, by integrating aspects of hospice culture (e.g., symbolic gestures involving candles), trainers could support emotional expression and communal processing (Running et al. 2008):

And then, of course, there are daily or weekly events such as when someone on the hallway is in palliative care. Then of course everyone who is in contact is naturally a bit shaken up. […] Or you might light a candle because someone has just passed away. (I1, T1)

#### 3.3.4 Reflexive Competencies During the Intervention

An essential aspect of trainers’ professional development involved self‐reflection on both programme‐related content and personal methodological competencies. These personal skills encompassed self‐organisation, trainer‐related expectations, self‐care, knowledge acquisition and (self‐)reflection, flexibility and willingness to learn.

As noted in Section 1.4 (‘Room Conditions’), trainers were required to arrive early at the LTC facility to prepare the training environment, highlighting the importance of self‐organisation in managing potential disruptions. Similarly, T3's decision to postpone the initial session due to insufficient information being communicated to participants reflects an expression of this competency.

All trainers reported varied expectations regarding the group context prior to the first session. While T1 and T2 had an opportunity to meet their groups during the recruitment and assessment phase, T3 and T4 had limited knowledge of their participants. Their primary concern centred on how participants would respond both to them as trainers and to the programme content:

So, my previous experience was limited to individual situations with older people, and I was very curious to see what the group situation would be like and how I would be accepted personally. (I1, T2)

The aspect of self‐care emerged in situations where trainers acknowledged their limits during the intervention. This included instances when participants’ seating posture hindered their participation in exercises, yet could not be modified by the trainers. Occasionally, a degree of emotional detachment was sometimes necessary to manage internal doubts arising from particularly stressful or challenging moments. For example, during T4's first session with an uninformed group, her empathetic response enabled her to perceive participants’ dissatisfaction and sense of overwhelm:

Um, yes, and then I just had to calm myself down a little bit and say, okay, you're going to make the best of it now. (I3, T4)

‘Expansion of knowledge’ referred to trainers’ reflection on and consolidation of the competencies they acquired through working with different groups. All trainers emphasised the value of leading group sessions aimed at maintaining participants’ swallowing, speech and perceptual abilities:

And yes, for me it was really […] this group situation, a total benefit and not only in terms of the instruction, but also in terms of dealing with disharmony in a group. (I1, T2)

The process of ‘self‐reflection’—closely linked to flexibility and a willingness to learn—encompassed trainers’ preparation, post‐session evaluation, adaptive thinking and critical examination of their own doubts, limitations and strengths:

It's difficult to respond to everyone individually, because it's a group setting. (I2, T3)

**FIGURE 4:**
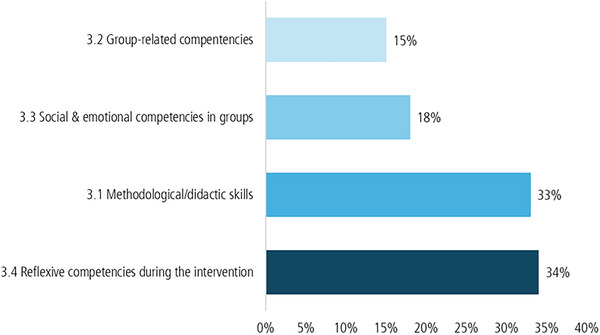
Code‐frequency of coding units for the subcategories of the first third category.

Figure 4 shows that methodological and didactic skills concerning the group programme exercises played a major role, particularly in group situations. However, self‐reflective skills became even more apparent in the interviews.

**FIGURE 5:**
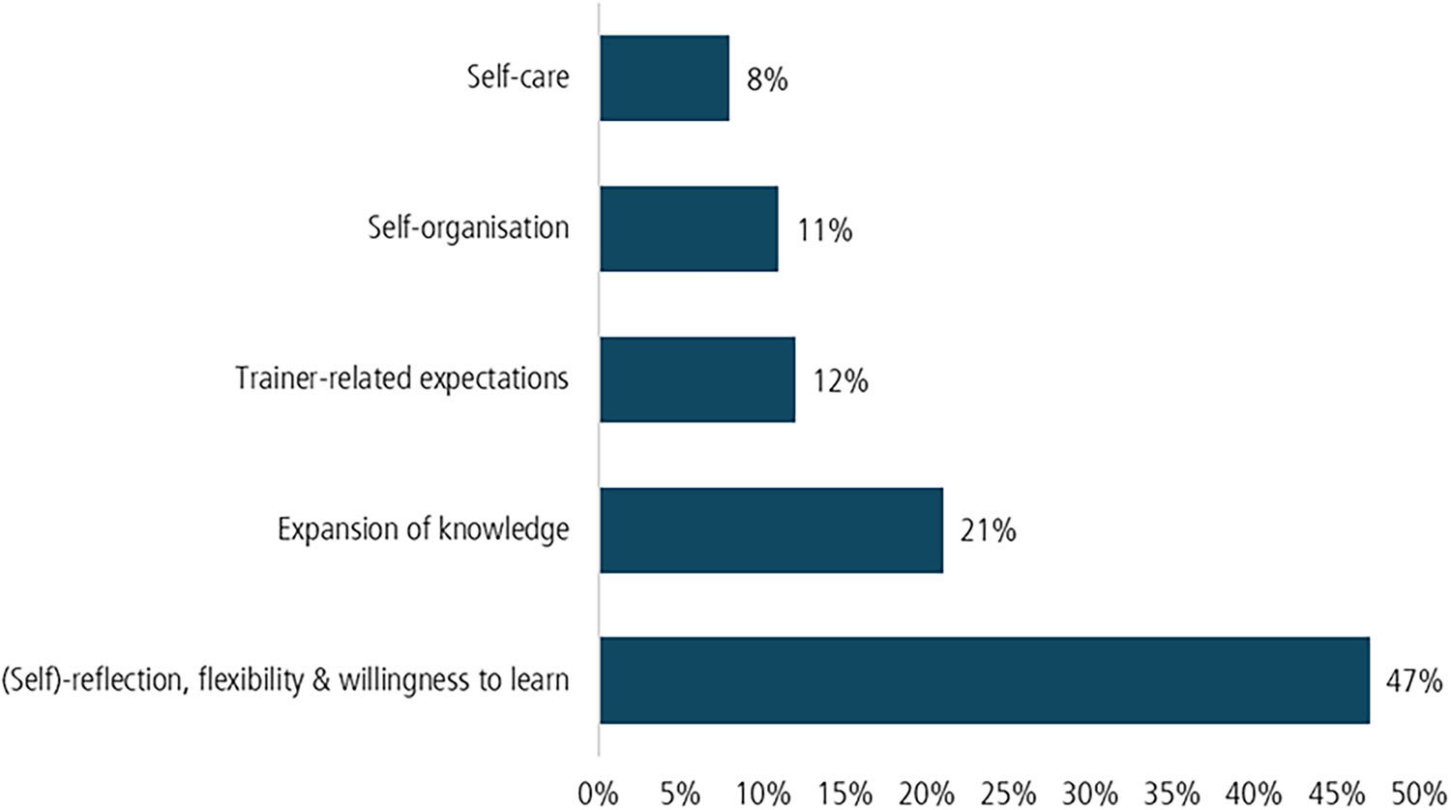
Detailed insight into the subcategory ‘Reflexive competencies during the intervention’.

Because the category of self‐reflective skills was coded so frequently, Figure 5 below provides a deeper insight into this category and the skills that trainers needed to carry out the intervention.

## 4 Discussion

The present findings revealed a multitude of factors influencing the implementation of preventive group training for older adults in LTC facilities. The three main categories emphasised the importance of specific trainer competencies in response to various external and internal influences. External factors included the structural and organisational conditions of the facilities and room environments, as well as the effectiveness of communication channels among stakeholders. Internal influences, on the other hand, comprised group dynamics, participant limitations due to illness or frailty, and group‐ or manual‐related challenges. Together, these elements contributed to the development of trainers’ self‐reflective, empathetic and problem‐solving competencies. Notably, external and internal factors did not operate in isolation, but interacted dynamically throughout the intervention process.

### 4.1 Promoting and Hindering External Factors

The findings concerning external influences highlighted the critical conditions required for successful group interventions in LTC contexts. While trainers consistently demonstrated empathy and sensitivity to participants’ needs and limitations, the effectiveness of the training programme also depended on institutional support to minimise workflow disruptions. This finding aligns with the existing literature, which stresses that both group therapy and training require targeted support to address participants’ individual challenges (Agronin 2009). In LTC facilities, such challenges frequently include mobility constraints affecting access to sessions, frequent toileting needs and the provision of assistive devices to accommodate visual and auditory impairments. Nursing personnel are critical to supporting these needs (Agronin 2009). As the interview data revealed, trainers were reliant to varying degrees on the support and engagement of their LTC facility. This is consistent with literature from the field of implementation science on the barriers and facilitators to implementing music interventions in nursing homes for people with dementia and depression (Rasing et al. 2025). In this literature, interventionists also cited a lack of support as one of the main barriers.

Moreover, the extent to which participants were informed about the programme in advance also influenced its success. Disruptions in programme flow were often linked to inadequate communication between facility staff and participants. The literature also highlights the advantages of clear communication and efficiently functioning teams and interest groups that actively foster and promote implementation (Rasing et al. 2025). The interviews suggested that insufficient information prior to the intervention could lead to confusion, emotional overwhelm and reluctance to engage. In line with this, previous research has emphasised that the provision of comprehensive information about the purpose and aims of a group intervention is a key success factor (Gephart 2013). Changes in employees and, consequently, contact persons were also assessed as a moderate influencing factor by those carrying out the interventions (Rasing et al. 2025). Hence, effective communication and coordination between trainers and LTC staff are crucial prior to the initiation of a programme.

As mentioned in the interviews, another important point is integrating the intervention into the daily schedule of LTC facilities. To this end it is also important that the intervention can be adapted to fit in with the facilities’ work processes (Fisher et al. 2025). In this regard, it may also be beneficial for the intervention to be carried out by an external person, in order to minimise the additional workload for the nursing staff (Rasing et al. 2025).

### 4.2 Group Characteristics (Promoting/Hindering)

Another significant aspect concerned the degree of homogeneity within the group. The interviews indicated that a certain level of uniformity—particularly in terms of cognitive and physical ability, as well as personality traits—was advantageous. Thus, trainers were responsible for addressing participant differences and developing effective strategies to manage them. As Hoppmann and Kessler (2024) argued, maintaining group homogeneity is vital to prevent any single individual from dominating the group dynamic. Additionally, Baron et al. (2018) notes that homogeneity ensures the group leader that no single participant receives excessive attention. Similarly, Agronin (2009) highlighted the importance of comparable cognitive functioning and ego capacity—encompassing perception, reasoning and memory—to facilitate meaningful participation. However, he also noted that individuals initially perceived as too impaired for group participation may, in some cases, engage successfully. This suggests that a certain level of heterogeneity is not only inevitable, but potentially beneficial, provided trainers are adequately equipped to manage such variation.

The present evaluation highlighted several challenges associated with heterogeneity in the participant group, including the need to adapt exercises and motivate participants with more pronounced limitations. According to the interviews, adjustments to the target group were necessary while maintaining the intervention's objectives. Some exercises had to be adapted for people with visual, hearing and/or cognitive disabilities (Davis et al. 2022). However, while group homogeneity is desirable, the presence of diverse abilities and backgrounds may also enrich the group experience by fostering personal growth and generating positive group dynamics (Gephart 2013). Achieving an appropriate balance between homogeneity and heterogeneity is difficult, and careful selection of participants is therefore recommended (Gephart 2013).

In addition, the motivation of the individual participants plays a significant role. As the interviews revealed, intrinsic motivation among participants is conducive to the success of group interventions. Conversely, a lack of motivation can hinder implementation. This aspect is also emphasised in literature on the implementation of music interventions in LTC facilities (Rasing et al. 2025).

Additionally, the literature on group interventions—particularly in psychotherapeutic contexts—points to a potential risk of stigmatisation among older adults (Elshaikh et al. 2023). In the specific case of the OrkA programme, which seeks to preserve speech, language and swallowing functions in older age, it may be essential to formulate the group's title, description and educational content in a manner that avoids reinforcing stigma, as age‐related cognitive and muscular impairments may elicit feelings of shame or insecurity. The interview data suggested that participants were often unaware of the programme's preventive focus. As a result, trainers had to reiterate the intervention's objectives and occasionally revisit the theoretical rationale concerning age‐related decline. However, this reignited participants’ pre‐existing fears and anxieties, particularly among those already feeling overwhelmed.

### 4.3 Therapeutic Competencies for Group Interventions

A further key consideration in group interventions with older adults is the decline in their abilities and motivation to perceive the thoughts, feelings and intentions of others. As Hoppmann and Kessler (2024) explained, the presence of a group leader is essential in overcoming these limitations and facilitating interaction among participants. However, this role carries considerable responsibility and demands a high level of empathy from trainers—a point substantiated by the present evaluation. The role of the trainer is multifaceted and closely linked to the experience of shame often reported among older adults, as discussed by Agronin (2009). This sense of shame may stem from the perceived loss of core aspects of personal identity. Consequently, in line with the existing literature on group dynamics and therapeutic processes (Agronin 2009; Hoppmann and Kessler 2024), trainer attributes such as empathy, acceptance, appreciation and the ability to respond sensitively within a group setting are crucial. A trainer working with cognitively impaired older adults must support the development of reorientation, memory and verbal skills in ways that do not evoke feelings of incompetence or embarrassment. Apart from the above aspects, group leaders should also understand varied behavioural goals associated with ageing (Agronin 2009).

In the context of group therapy, Gephart (2013) underscored the importance of experiential learning as foundational for practitioners in the psychotherapeutic field. Such learning—grounded in real‐life group experience—cannot be fully acquired through theoretical instruction alone. Accordingly, familiarity with the different phases of group development is essential. The classic model of group progression—*forming*, *storming*, *norming*, *performing* and *reorientation* (Wellhöfer 2018)—provides a theoretical framework and practical foundation for group facilitation. Each phase of this model encompasses not only tasks for the individual participants but also responsibilities specific to the group leader.

For instance, the *forming* phase is particularly sensitive, as participants typically experience heightened insecurity. The primary aim during this stage is to establish a basis for mutual acquaintance, introduce the training topic and build initial trust in the trainer. The trainer should therefore foster a relaxed atmosphere and allocate dedicated time for small‐group dialogue (Wellhöfer 2018). The interview data confirmed that the initial session played a pivotal role in the intervention trajectory. However, despite trainers’ preparation and reflective capacities, various external factors influenced the process. This presented a considerable challenge for group trainers, highlighting the importance of practising and supervising interventions within a structured training context. In relation to the *forming* phase (Wellhöfer 2018), the data suggested that participants actively engaged in the process of getting to know one another, forming subgroups, and—in most cases—establishing a sense of positive group cohesion.

Cohesion may be particularly beneficial for older adults affected by loneliness (Hoppmann and Kessler 2024), in which case the *storming* phase (Wellhöfer 2018) will hold particular significance in relation to trainer competencies. In the present intervention, trainers had to create opportunities for group members to express both strengths and vulnerabilities, encouraging manageable rivalries that could be addressed within a safe and structured environment (Wellhöfer 2018). The interviews showed that this was a demanding stage for trainers. Establishing clear conversational guidelines was essential, by fostering mutual understanding, supporting inclusive dialogue and navigating challenging group dynamics while engaging in ongoing self‐reflection. This is in line with literature on previous communicative interventions, which states that group leaders are responsible for facilitating conversational interactions between group members during interventions with older adults in LTC facilities. It is recommended that initiative can be demonstrated by all participants (Davis et al. 2022).

Furthermore, the trainer must be able to deal with and respond to any unexpected problems that arise within the group (Baron et al. 2018). As the interviews revealed, some participants were rather quiet and contributed less to group discussions. The trainers had to respond encouragingly and select activities that ensured the involvement of all participants without compromising the comparability of the intervention. Additionally, a lack of motivation or disinterest among participants can pose a challenge that must be addressed. To this end, the trainers had to repeatedly emphasise the aim of the group intervention. This approach is consistent with references in the literature (Baron et al. 2018).

Trainer responses had a notable impact on group dynamics; nevertheless, the overarching objective of the intervention remained paramount. These findings suggest that both constructive and destructive group processes—including the emergence of collective dynamics beyond individual contributions—should be integrated into professional education programmes (Gephart 2013).

The challenges encountered during the OrkA sessions strongly support the inclusion of group therapy within the training curriculum for SLTs, thereby preparing future professionals for group‐based practice. However, the implementation of such group formats requires specific structural conditions, such as those outlined by Ries (2022) for physiotherapists working with individuals with dementia. Ries (2022) also underscored the centrality of relationships and communication between therapists and participants—a point echoed in the present evaluation. Indeed, the interview findings confirmed that communicative competencies were vital for stakeholder coordination, effective information exchange and the development of therapeutic relationships between trainers and participants. These results are consistent with the literature, which identifies communication and collaboration as critical to the success of interventions (von der Warth et al. 2021).

Overall, the interview findings reinforced core aspects of psychological group therapy and underscored the importance of group‐related therapeutic competencies in the field of SLT. Regrettably, the current German curriculum for SLT includes little to no content on group work (Bundesamt für Justiz 1980).

### 4.4 Strengths and Limitations

The present analysis provided comprehensive insights into a preventive intervention programme for older adults in German LTC facilities. This programme, which currently has no equivalent, offers a distinctive perspective on the specific challenges and requirements of this demographic. The interviews also yielded valuable information concerning potential modifications of the programme for future establishment as standard care. The timing of the interviews—conducted immediately following the intervention period—proved advantageous, enabling the collection of rich, detailed accounts. The data saturation supports the robustness of the findings. Furthermore, the exploratory nature of the qualitative research facilitated the generation of new knowledge (Perkhofer et al. 2023) in a relatively underexplored area of healthcare. The evaluation also focused on group‐based practical work—a domain in which SLTs are seldom consulted, thereby adding to the field's emerging evidence base. Complementing the quantitative design of the OrkA RCT, the present interview data offered valuable experiential insights to enhance and contextualise the quantitative results. However, the findings were constrained by the extent of the trainers’ willingness and ability to recall and articulate their experiences. Additionally, the collegial relationship between the interviewers and the interviewees may have introduced biases. As previously stated, there is a possibility of selection bias due to the absence of interviews with other relevant parties, despite the trainers, who were also members of the research team. Interviewing other parties involved in the intervention, such as management or employees of the institutions, would have provided additional opinions and perspectives. These would also have been valuable for evaluating the intervention. The residents' perspective was also considered interesting, so their satisfaction was evaluated. Due to the volume of data, the results were published in a separate paper (Walther and Fesser 2025). Finally, the findings were derived exclusively from the OrkA project and thus limited to the perspectives of the four participating SLTs (trainers). While this restricts generalisability, it also serves as a strength: the trainers’ detailed reflections on the skills they developed and their capacity to flexibly adapt the material contributed significantly to the depth of the analysis. Thus, although the findings cannot be extrapolated to other settings without caution, they may still hold relevance for professionals in other disciplines offering group‐based interventions within LTC environments. It is important to note, however, that the conclusions pertain specifically to a preventive intervention model. Discrepancies may arise when comparing these outcomes with those of therapeutic group interventions based on different theoretical or clinical approaches.

## 5 Conclusion

The OrkA group intervention—evaluated through a cluster RCT—represents a preventive approach aimed at maintaining swallowing and speech abilities in older adults. This intervention, currently without equivalent in Germany, adopts a multifaceted framework addressing external and internal factors, as well as their interrelationships. Based on the interview data, the following key conditions were identified for the successful implementation of group interventions aimed at preserving swallowing, speech and communication abilities in older adults in LTC settings:

− Institutional context: the general structure and organisation of the intervention within LTC facilities;
− Group composition: balancing homogeneity and heterogeneity;
− Skills and attributes of the trainers:
− Trainers’ methodological skills in responding to individual needs; and
− Trainers’ group management skills, which should be developed during practical training.

Indeed, the role of the trainer emerged as central to the success of group training with older individuals. Trainers must simultaneously assume the role of facilitator, listener, confidant, emotional supporter, motivator and mediator—in relation to both the intervention content and interpersonal group dynamics. A high degree of external disruption (e.g., inadequate communication among stakeholders, unsuitable room conditions, unclear organisational arrangements) could significantly affect group dynamics. In such instances, trainers had to demonstrate problem‐solving competence and flexibility. Further research is needed to better define the competencies required of trainers leading group interventions in both therapeutic and preventive contexts. Nonetheless, based on the present findings and in light of health insurance requirements concerning therapist preparation for group‐based formats, it appears both appropriate and feasible to incorporate group training into the education of SLTs. Overall, the present findings offer valuable insights for future research and the development of structured frameworks to guide therapists and caregivers in delivering group training interventions for older adults.

## Funding

The study was funded by the Innovation Fund of the Federal Joint Committee (Innovationsfonds des Gemeinsamen Bundesausschusses, Grant No. 01VSF22043). The funders had no role in the study design, the data collection, the data analysis, the programme evaluation or the interpretation of the quantitative or qualitative data. They did not contribute to the writing of this report, nor did they influence the decision to submit the manuscript for publication. Open Access funding was enabled and organised by ‘Projekt DEAL’.

## Ethics Statement

The OrkA RCT was approved by the Ethics Committee of Hannover Medical School Germany on 2 February 2023 (no. 10650_BO_S_2022). The Ethics Committee also reviewed and approved the interviews conducted as part of the process evaluation.

## Consent

Written informed consent was provided by all participants and interviewees prior to any study‐related activity. The study was conducted in accordance with the Declaration of Helsinki.

## Conflicts of Interest

The authors declare no conflicts of interest.

## Supporting information




**Supporting Information**: jlcd70226‐supp‐0001‐SuppMat.docx

**Supporting Information**: jlcd70226‐supp‐0002‐SuppMat.docx

**Supporting Information**: jlcd70226‐supp‐0003‐SuppMat.docx

## Data Availability

The anonymised transcripts supporting the study findings are available from the corresponding author upon reasonable request.
