# Supplementary material for: Qualitative Insights on Preventive Group Training in LTC Facilities: Key Influencing Factors
Source: Int J Lang Commun Disord. 2026 Mar 19;61(2):e70226. doi: 10.1111/1460-6984.70226 (PMC13002556; doi:10.1111/1460-6984.70226)
Supplement: Supplementary file 1 — Supporting Information: jlcd70226‐supp‐0001‐SuppMat.docx [file JLCD-61-0-s003.docx]

**Guidelines for interviews with trainers of the OrkA intervention**

**Welcome and consent** –

Thank you very much for agreeing to participate in this interview and for taking the time today. You have already received written information about consent and data protection. Do you agree? Is there anything you would like to add or ask?

**Procedure** – I/we would like to talk to you about your experiences with the programme. This will help us to adapt it further. I/we have prepared a few questions for you. Please tell me/us what comes to mind. My/our job is to listen to you and ask questions if I/we don’t understand something or need more information on a topic.

**Audio recording** – With your permission/agreement I/we would like to record our conversation so that I/we can focus on our discussion and ensure that nothing is missed.

**Anonymity** – Everything you say will, of course, be treated confidentially. When transcribing the conversation, we will make sure to remove names and other details, such as places or institutions, so that no conclusions can be drawn about the care home, individuals, or your specific situation.

**Time** – Based on my/our experience, the interview will take about 1 hour. However, I/we would be happy to adapt to your needs. At any time, let me/us know, if you are unable to continue talking or if you need a break. You can also ask me/us to turn off the tape recorder.

| **Story prompts/guiding questions** | **Memo/checks** | **Follow-up/in-depth questions** |
| --- | --- | --- |
| **1. General experiences with the intervention**  Could you **start by** telling me about your personal experience and your general impression of the situation at the facility and in the group intervention? | Relate your own situation and setting | - *Group processes* - *Interaction with the facility (staff)* - *Your own previous experiences* - *Own fears beforehand* |
| **2. Course of the intervention**  **2.1 Organisation**  What was your experience in terms of time and spatial organisation?  Please tell us what it was like. | Organisation/ time frame | - *Duration of individual sessions* - *Duration of 12 weeks* - *2x per week* - *Time of day in the morning* - *Room conditions* |
| **2.2 Content: Mobilisation**  What did you experience during the mobilisation exercises?  What were your thoughts on this part?  Which exercises did you choose? | Mobilisation | - *Reflect on your own choice* - *Group reference* - *Feasibility* - *Relevance* |
| **2.3 Content: Swallowing exercises**  What was your experience carrying out the swallowing exercises? What did you think of them? | Swallowing exercises | - *Relevance to the ChinTuck exercises* - *Feasibility* - *Difficulty level* |
| **2.4 Content: Semantic exercises**  What was your experience with the language and word-finding exercises? What did you think of the exercises? | Semantic-lexical part | - *Feasibility* - *Interest appropriate* |
| **2.5 Content: Biographical conversation**  How did you experience the biographical conversations? What were your experiences? | Interview/Conversational sequence | - *Relevant* - *Sufficient time* - *Distribution of speakers* |
| **2.6 Content: Conclusion/Closing song**  What was your experience with the closing song? | Song | - *Selection* - *Own abilities* - *Relevant* - *Acceptance* |
| **3. Material**  You have already talked about the material:  Are there any other experiences you have had with using the material that we have not yet discussed? | Material | - *Didactics* - *Appealing?* - *Swallowing exercises* - *Semantic-lexical exercises* - *worksheets for the voluntary homework (VHW)* - *Worksheets* - *Preparation folders with the material for each session* - *Binder fort he participants* |
| **4. Benefits**  What is your personal impression? Did the intervention benefit the residents? | Individual benefits | - *Visible improvements (swallowing/language related aspects)* - *Residents‘ feedback* |
| **5. Final questions**  **5.1 Prevention**  All things considered, what do you think would be needed to preventively improve speech and swallowing in seniors? | Perspective | - *Other clientele* - *Occupation of trainers* |
| **5.2** What would be important in the application of the OrkA programme? |  |  |
| **5.3** Is there anything that is important to you but has not been addressed? | Additions |  |
| **Thanks**  Thank you very much for taking the time to talk to me/us! You have given me/us a lot of valuable feedback! | | |
