# Supplementary material for: Qualitative Insights on Preventive Group Training in LTC Facilities: Key Influencing Factors
Source: Int J Lang Commun Disord. 2026 Mar 19;61(2):e70226. doi: 10.1111/1460-6984.70226 (PMC13002556; doi:10.1111/1460-6984.70226)
Supplement: Supplementary file 2 — Supporting Information: jlcd70226‐supp‐0002‐SuppMat.docx [file JLCD-61-0-s001.docx]

**Additional information about the interviews**

Trainer T1 was interviewed three times in total, while trainer T2 was interviewed once and trainers 3 and 4 were interviewed twice. The difference in the number of interviews is explained by the fact that T1 had the most intervention groups (6) and T2 delivered just one group.

The interviews were always supposed to be conducted by two researchers and one trainer. However, due to circumstances such as appointments and illness, it was sometimes necessary to deviate from this.

In the first interview (I1), a tandem interview was conducted with T1 and T2. Both researchers (WW and SM) and a person taking minutes were also present. The first and second interviews with T3 (I2 and I5) were also conducted by WW and SM. The first and second interviews with T4 (I3, I6, were conducted by WW. The second interview with T1 (I4) was conducted by SM. The third interview with T1 (I7) was conducted with both WW and SM.

| Interview number | Number of researchers | Number of trainers | Number of reported interventions | Number of note takers |
| --- | --- | --- | --- | --- |
| I 1 | 2 (WW, SM) | 2 (T1, T2) | 3 | 1 |
| I 2 | 2 (WW, SM) | 1 (T3) | 1 | 0 |
| I 3 | 1 (WW) | 1 (T4) | 1 | 0 |
| I 4 | 1 (SM) | 1 (T1) | 2 | 0 |
| I 5 | 2 (WW, SM) | 1 (T3) | 1 | 0 |
| I 6 | 1 (WW) | 1 (T4) | 1 | 0 |
| I 7 | 2 (WW, SM) | 1 (T1) | 2 | 0 |
