# Supplementary material for: Qualitative Insights on Preventive Group Training in LTC Facilities: Key Influencing Factors
Source: Int J Lang Commun Disord. 2026 Mar 19;61(2):e70226. doi: 10.1111/1460-6984.70226 (PMC13002556; doi:10.1111/1460-6984.70226)
Supplement: Supplementary file 3 — Supporting Information: jlcd70226‐supp‐0003‐SuppMat.docx [file JLCD-61-0-s002.docx]

|  | Category  designation | Subcategories | Coding rule |
| --- | --- | --- | --- |
| MC 1 | **Promoting or hindering external factors**  The first main category refers to the in-depth questions relating to the first two sections of the interview guide, which cover general experiences of the intervention and organisation. It is based on the research interests of the process evaluation and was therefore derived deductively from the guide.  The focus is on general experiences and factors that are described as potentially promoting or hindering the implementation of training, and thus its success. Whether the trainers explicitly describe the aspects themselves as promoting or hindering training is irrelevant. Even the mere suggestion that a factor is positive, beneficial or motivating, or negative, demotivating or otherwise detrimental, is sufficient for coding.  **Please note** that categories are often mixed together in the material, and the respective weight of the category must be identified during coding. When coding, it is often necessary to decide in favour of one of two or three overlapping categories.  **Research question:**  Under what conditions is the intervention successful? Which inhibiting and promoting factors influence the success of the intervention? | - **SC 1.1 Start at the facility**   **(+/-)**  This category was derived deductively from the interview guide. This question was asked at the start of each survey. | **(+)** This category should be coded when trainers report that they received a warm and friendly welcome from the institution at the start of their training. This would be the case, for example, if they said that the people who welcomed them knew their start date and could competently introduce them to the institution.  It is also relevant if trainers were treated in a manner that made them feel safe at reception. The category is also relevant if the trainers was immediately shown to the training room and introduced to the premises competently. The use of adjectives such as positive, good or warm is irrelevant. Even hints at these facts are sufficient for coding this category.  **(-)** This category should be coded if trainers report that they received a poor welcome from the facility at the start of the training. This would be the case if they said that the facility was unaware of the start of the training.  The same applies if trainers were treated in a manner at reception that could have caused them to feel uncertain. The category is also relevant if the institution did not immediately show the trainers to the training room and the premises were not clearly organised. The use of adjectives such as 'negative' or 'unfortunate' in the coding unit itself is irrelevant. Even hints of these circumstances, such as the words "actually" or "somehow", are sufficient for coding this category.  **Distinction rule:** If there is mention of unclear room conditions on the first day, this belongs in category 1.1. If the issue is room changes during the programme, select category 1.5. |
|  |  | - **SC 1.2 Communication, information and agreements**   **(+/-)**  This category was derived from the interview guide using a deductive approach. Questions about interaction with the facility were asked in each interview. | **(+)** **Communication:** This category should be coded if the trainers report that communication and agreements between the participants and the central training coordinators were successful. This would be the case, for example, if the institution had been informed about the training, the trainers and their requirements. All communication channels between the institution, training developers, trainers and participants fall into this category.  **Information:** This is particularly the case when participants are informed about the content and background of the training, the trainers, and what to expect, including the dates of the group sessions.  **Agreements:** This also applies when appointments or similar matters were easily arranged between the trainer and the institution or the trainer and the participant.  **Distinction rule**: If the trainers said that they were well received by the institution, then category 1.1, 'Start at the facility', is relevant, not this category.  **(-)** **Communication:** This category should be selected if the trainers report unsuccessful communication and coordination between the various participants and the central training hubs.  **Information:** This is also the case if participants were not informed about the training (including its content), the start date, or the trainers, and were unaware of what to expect or when the training sessions would take place.  **Agreements:** All unsuccessful agreements (including appointments) between the institution, training developers, trainers and participants during the training course fall into this category. If trainers report that agreements were not kept, then this category is relevant.  **Distinction rule:** If the trainers say that they were not well received by the institution, then category 1.1 Start at the facility is relevant, not this category. However, if it concerns pre-communicated knowledge between the institution, training developers, trainers and participants, this category should be coded. |
|  |  | - **SC 1.3 Whole organisation and support (+/-)**   This sub-category was derived deductively, as it relates to the second major section of the interview guide. | (+) **Overall organisation is unproblematic:** This includes the fact that the intervention and its timeframe can be seamlessly integrated into the institution's structure without overlapping with other programmes, enabling all participants to engage fully.  Therefore, it is a matter of organisational framework conditions, ensuring that the intervention as a whole can take place without barriers within the respective institution.  Support: Select this category if the institution provided support. This includes the provision of resources (e.g. water, straws, spoons, glasses) and personnel support (e.g. bringing and collecting participants). Support can also refer to staff helping with homework if participants are unable to read the tasks independently.  **Training process:** This is interwoven with statements about an undisturbed training process. If trainers report that the training was carried out well due to appropriate support from the home, then this category is relevant. This includes the participants having had breakfast with the help of facility staff and being in the group room on time at the start of the training.  (-) **Problematic overall organisation:** Difficult integration of the intervention into the facility's processes and poor organisation on the part of the home. If the time slot overlaps with other home services, participants may not participate in the intervention, or may only participate once a week.  Information that participants attended despite other services being offered, but found it stressful (because they had to decide each time), should also be coded under this category.  If trainers report that the time slot had to be changed at short notice, leading to cancellations or near-cancellations, this category should also be selected.  **Lack of support:** Select this category if there were facility-related obstacles during training due to a lack of support. This includes participants in need of care who were not ready on time, were not made ready, and were still in bed in their pyjamas; or who were unable to have breakfast on time due to a lack of blood sugar measurements, meaning they were unable to attend the training at all, or could only attend later (interruptions). |
|  |  | - **SC 1.4 Room conditions (+/-)**   This category was also derived from the interview guide using deductive approach. Room conditions were a major theme that was covered in each interview. | (+) **Optimal:** This category should be selected if the training room was described as ideal, bright, open, usable or functional by the trainers in the coding unit. This includes descriptions of the training room being ideal, bright, open, usable or functional, for example. The category is also relevant if the interviews mention that the training room was well structured and designed for training purposes, or if the trainers found their way around the premises easily and did not have to change rooms. The category should also be used if the trainers mention that they were able to work in the training room without interruption because it was reserved exclusively for OrkA training purposes.  (-) **Suboptimal:** This category should be coded if the trainers in the coding unit mention that the training took place under suboptimal spatial conditions. This includes descriptions of the training room as dark, small, cold or untidy, for example. The category should also be coded if the interviews mention that the training room was unstructured or not prepared for training needs, or if the trainers found it difficult to find their way around the premises or had to tidy up first.  The category should also be coded if trainers mention that they were unable to work continuously in the training room because the institution did not reserve it exclusively for OrkA training purposes.  This category also addresses whether the allocated room needed to be adapted to carry out the intervention (e.g. tables that were missing or too small). Information about noise levels or glass doors that distracted participants should also be assigned to this category. |
|  | **MC 2 Group characteristics (promoting/hindering)**  **(+/-)**  This main category was derived inductively.  The selection criteria were defined as follows:   - Statements in which trainers discuss the composition of groups (i.e. their heterogeneity or homogeneity) - Statements in which trainers discuss frailty and special needs - Statements in which trainers discuss influences on the intervention (+/-) - Statements in which trainers discuss special situations due to participant requirements - Statements in which trainers discuss the absence or presence of mutual interaction | - **SC 2.1 Beneficial or hindering group characteristics**   This category was derived inductively. The interviewers did not directly ask about beneficial or hindering aspects. Some questions were posed with reference to the group. Especially when the time frame and the different exercises, voluntary devices and written materials were considered. | **Beneficial:**  Conducive characteristics: This category includes intrinsic motivation, performance level, and interests and curiosity. If these characteristics are comparable within the group, the respective coding units are assigned to this category. Statements about active participation, questions of interest and understanding of the project background within a group are also assigned to this category.  Independence: Participants were highly independent and well organised, able to arrange other appointments around the programme, organise their mornings to arrive at the training room on time and meet without a group leader. It also includes having all the necessary materials (folders, sheets of paper, etc.).  **Hindering:**  This category assesses the extent to which participants were able to organise themselves and prepare independently for the sessions. This includes statements about missing materials, frequently forgetting the folder, misplacing the folder and a lack of structure within it. If the trainers reports that folders and/or necessary materials were unavailable, could not be found, or were forgotten, this category should be selected. Information about problems filing sheets is also coded in this category. Statements about the groups being dependent on their trainers and needing them to come together as a group are also important. |
|  |  | - **2.2 Participants with specific challenging needs**   This category was derived inductively. The trainers provided detailed explanations of various participants with exceptional needs that go beyond the usual differences between participants. | Select this category if there are general statements about a high level of frailty (cognitive, physical, etc.) among participants in a group. It also includes references to irregular participation or disrupted proceedings due to frailty. Statements about dropouts due to excessive cognitive demands should also be assigned to this category. If the trainers report a strikingly heterogeneous group with 'frail outliers', this category should be selected. |
|  |  | - **2.3 Anxious group members**   This category was derived inductively. It is different to participants with specific challenging needs, which were related to diagnoses. Anxious group members have been described differently in the material. | This category should be selected if the trainers reports that participants in their group are frightened, scared, sceptical or worried. It is irrelevant whether these fears and concerns existed prior to the intervention or arose as a result of it addressing swallowing and speech problems. |
|  |  | - **2.4 Social and interpersonal aspects**   This category was derived inductively. Some groups had no mutual understanding, while others were quite sociable with one another. These differences led to different needs emerging during the interviews. | This category should be coded if the trainers report positive group dynamics and harmony, and strong group cohesion. Topics covered include mutual respect, appreciation, adherence to communication rules, and acceptance. Interview passages indicating patient and appreciative interaction among participants (e.g. everyone was given an equal opportunity to speak and no one was criticised negatively) are coded in this category.  Conversely, this category should not be used when the trainers report poor group dynamics/harmony and a lack of group cohesion. This includes issues such as a lack of respect, understanding and appreciation. Interview passages indicating impatient or disrespectful behaviour towards one another (e.g. participants interrupting each other, negative criticism of individuals and unequal sharing of needs) are coded under this category. |
| MC 3 | **Therapeutic competencies for group interventions**  The third main category was derived deductively and inductively from the material. It emerged during the interviews when the feasibility of various content areas (mobilisation, semantic, swallowing exercises and conversation) was discussed. Additionally, some follow-up questions in different sections of the interview guide raised this topic at various levels, including methodological and didactic, group-specific, and individual therapist levels.  Top category 3 of the category system focuses on all factors relating to the outstanding competencies required by trainers to carry out the group intervention. Particular emphasis is placed on group-related competencies necessary for managing the group and dealing with group dynamics and heterogeneity. Statements relating to communication and interaction within the group are relevant. In this context, trainers' self-reflection, uncertainties, and ideas for solutions are also highlighted.  **Research question:** What competencies are needed by trainers for the successful implementation of the intervention? | - **3.1 Methodological and didactic skills**   This category was derived deductively, as the exercise reflections formed a significant part of the interviews. These questions were always posed in reference to the group to determine feasibility. The interviewers also asked about alternative exercises when the manual was not followed.  **Research question:** What methodological competencies, skills and abilities are required of speech therapists working in preventive group settings, using OrkA as an example? | **Didactic skills:** This category includes interview passages in which trainers discuss didactic aids. Examples include the use and marking of homework, progress checks, precise demonstrations by the trainers and suggestions for imitation and comparison. The use of educational sequences in which the trainer provides additional information on individual exercises to further explain their purpose (e.g. by providing further details on exercise-specific motor/muscular mechanisms) should also be coded in this category.  Problem-solving skills: Methodically adapting content, exercises and procedures to address forgetfulness, disorientation, physical limitations and individual preferences, and responding in a way that enables individuals to achieve the best possible outcome in terms of the project idea (i.e. providing guidance and support).  **Orofacial exercises:** Adaptations of orofacial exercises🡪 Intensity/severity/aids/rejection/OFS material  **Semantic exercises:** Adjustments to speech exercises🡪 Severity/speech system over- or under-challenge/material  **Discussion and conclusion:** Adjustments to the last two sequences🡪 Discussion/conclusion/alternatives |
|  |  | - **3.2 Group-related competencies**   This category was derived inductively, as these themes were not prompted by the interviewers, but were frequently mentioned during the interviews.  Selection criteria:   - Statements in which trainers discuss specific situations involving misunderstandings within the group - Statements in which trainers discuss challenges concerning individual group members and the amount of attention they require - Statements in which trainers discuss very shy members and unilateral communication through extroverted members - Statements in which trainers discuss very emotional situations, such as when some groups had to manage themes like death or illness - Statements in which trainers discuss the lack of group unity at first glance - Statements in which trainers discuss very challenging situations in which individual team members had to deal with criticism or ongoing conflicts | This category focuses more on verbal group management.  It covers **controlling group dynamics and cohesion**. It includes statements about strengthening and creating group cohesion, as well as the need to establish mutual understanding. It also includes ideas for addressing certain group processes. It also involves managing conversations, for example controlling, steering, moderating and ending them.  **Conversation rules/manners:** Information about establishing conversation rules, as well as thoughts on 'how the group wants to function', are relevant to this category. Information about how to treat each other with respect and appreciation also belongs in this category. Respectful communication in challenging, critical situations.  **Individual vs. group:** Context units that deal with balancing responding to individual participants with special needs while considering the entire group are coded with this category. This therefore concerns information about dealing with different individual needs and performance levels within the overall group (it is difficult to get the whole group on board and understand the separate impact on individuals). Situations that require special handling include when individuals leave the group, refuse to do exercises or find them too difficult or too easy. These situations should not affect the group as a whole if possible. Situations where participants refuse to do exercises, become annoyed, or stop in order not to endanger the group also fall into this category.  **Distinction rule:** This refers to the reactions/actions of the trainer. If there is little understanding among the group, this is a hindering group characteristic (category 2.1).  **Promoting interaction within the group** involves providing guidance on mutual support and communication among participants. Encourage (quieter participants) to express their opinions, feelings or needs. The aim is to involve all participants. More verbal group control is required.  **Empathetic reactions:** Supporting individuals to enable them to contribute to the group should be assigned to this category (e.g. encouraging individuals to recognise that they are an integral part of the group, despite their 'frailty' or pre-existing conditions). Motivational speeches should be used to encourage active participation. This category includes interview passages in which trainers express their empathetic and sensitive decisions or approaches (e.g. when they report that they limited the extent of their criticism of exercise performance to avoid shame, or responded encouragingly to participants' discomfort and uncertainties).  **Constructive handling of tensions, criticism and conflicts:** Sometimes, de-escalation strategies such as one-on-one conversations or open discussion/questioning are employed in conflicts/tensions between participants. Information about how the trainers remained impartial is also relevant. It is also relevant to consider how to react to participants suggesting solutions and criticising the performance of others (e.g. 'that's totally easy'). Information about how to discuss constructive criticism in the event of inappropriate criticism is also relevant.  Delimitation rule to 3.3: This category concerns active responses. |
|  |  | - **3.3 Social and emotional competencies in groups** - Statements in which trainers demonstrate therapeutic qualities such as acceptance, empathy and sensitivity | This category focuses more on descriptions, i.e. what the trainers perceived and observed. It is not about any resulting action.  **Empathy and sensitivity:** The ability to perceive the needs, feelings and limits of the participants. Adapting the approach to the emotional and physical needs of the participants. Controlling the emotional aspect of the interaction (giving participants space to talk when they do not "dare").  **Acceptance and appreciation:** Respecting individual limitations and abilities. This category is not specifically about modifying certain exercises, but rather about dealing with heterogeneity, refusal to participate in exercises, uncertainties and overcoming obstacles in a therapeutic and accepting manner. This includes situations where participants decide not to perform certain exercises in any area.  **Sensitivity to group dynamics:** Recognising tensions, the formation of subgroups, the isolation of individuals, and a lack of mutual understanding. Initially, this involves simply describing such tensions, as this indicates a sensitive perception of group dynamics (e.g. when a therapist reports that there were two groups of friends and one individual in their group).  **Distinction rule:** Descriptions of perception and observation are relevant; this is not about specific actions. These would be coded with 3.2. |
|  |  | - **3.4 Reflexive competencies during the intervention**   This category was derived both deductively and inductively. The self-reflective starting point is deductive, as it formed part of the interview guide and was asked in every interview.  The second, third and fourth parts are inductive. These categories resulted from questions about the exercise adaptations.  Selection criteria:   - Statements in which trainers discuss or demonstrate reflection and learning - Statements in which trainers discuss or demonstrate changes in knowledge across intervention groups - Statements in which trainers discuss stress, emotions and demonstrate strategies like professional boundaries | **Trainer-related expectations:** These relate to individual settings, and groups present a new challenge. Fears: Concerns about how the group will react to the trainer conducting the session, and whether critical questions will be asked, should be assigned to this category. Thoughts: These include thoughts about the new setting for the trainers, such as working with a group of older people rather than doing individual therapy.  **(Self)-reflection, flexibility and willingness to learn:** This aspect includes the trainers' personal thoughts, fears and previous experiences. Reflecting on the sessions in order to derive new ideas that go beyond the manual. Recognising one's own limitations and challenges during the group intervention. Information about the necessary preparation and follow-up for the individual sessions (type, time required and individual exercises) also falls into this category.  Self-reflection can refer to personal skills, such as statements about how the trainers relied on and trusted themselves, did not allow themselves to be dissuaded from their goals and were able to deal with certain challenges over time.  **Expansion of knowledge:** Statements about participants acquiring new knowledge. This may be because the group had explicit questions or because certain knowledge gains resulted from the group training. References to self-reflective competence or knowledge expansion during the intervention belong to this category.  **Self-organisation:** This category is selected when the trainers demonstrate a certain degree of self-organisation. This includes instances where the trainers independently searched for missing materials, took care of other rooms or tidied them up for training purposes.  **Self-care:** Another competence relates to the trainers' ability to distance themselves from the participants' emotions, attitudes, opinions and criticisms to a healthy extent and at the appropriate moment, thus enabling them to take care of themselves. |
